# p38 MAPK Inhibition Mitigates Hypoxia-Induced AR Signaling in Castration-Resistant Prostate Cancer

**DOI:** 10.3390/cancers13040831

**Published:** 2021-02-17

**Authors:** Serina Cheung, Pallavi Jain, Jonathan So, Saeid Shahidi, Stephen Chung, Marianne Koritzinsky

**Affiliations:** 1 Princess Margaret Cancer Centre, University Health Network, Toronto, ON M5G 2C1, Canada; serina.cheung@mail.utoronto.ca (S.C.); Pallavi.Jain@uhnresearch.ca (P.J.); jo.so@mail.utoronto.ca (J.S.); saeid.shahidi@uhnresearch.ca (S.S.); stephen.chung@uhnresearch.ca (S.C.); 2 Institute of Medical Science, University of Toronto, Toronto, ON M5S 1A8, Canada; 3 Dana-Farber Cancer Institute, Harvard Medical School, Boston, MA 02215, USA; 4 Department of Radiation Oncology, University of Toronto, Toronto, ON M5T 1P5, Canada; 5 Department of Medical Biophysics, University of Toronto, Toronto, ON M5G 1L7, Canada

**Keywords:** castration-resistant prostate cancer, hypoxia, androgen receptor, p38 MAPK, Hsp27, xenograft, prostate specific antigen, SB203580

## Abstract

**Simple Summary:**

Progression of prostate cancer to a castration-resistant state is associated with poor patient outcomes, and new therapeutic targeting approaches are needed. Poorly oxygenated (hypoxic) cancer cells are resistant to many treatment modalities, and it is therefore important that novel therapies also target these cells. Here we show that targeting the p38 MAPK protein kinase can inhibit growth and survival of both well-oxygenated and hypoxic castration resistant prostate cancer cells and prolong survival of tumor bearing mice. p38 MAPK targeting inhibited phosphorylation of the chaperone protein Hsp27 and activity of the androgen receptor. This demonstrates that prostate cancer cells can remain dependent on the p38 MAPK/Hsp27 signaling axis upon progression to castration-resistance, and that hypoxia does not offer protection against targeting this pathway.

**Abstract:**

Background: Aberrant androgen receptor (AR) signaling is a major driver of castration-resistant prostate cancer (CRPC). Tumor hypoxia increases AR signaling and is associated with treatment resistance in prostate cancer. Heat shock protein 27 (Hsp27) is a molecular chaperone that is activated in response to heat shock and hypoxia. Hsp27 has previously been reported to facilitate AR nuclear translocation in a p38 mitogen-activated protein kinase (MAPK) dependent manner in castration-sensitive prostate cancer cell lines. Here, we evaluated the potential for inhibiting p38 MAPK/Hsp27 mediated AR signaling under normoxia and hypoxia in experimental models of CRPC. Methods: We inhibited p38 MAPK with SB203580 in prostate cancer cell lines and measured Hsp27 phosphorylation, AR activity, cell proliferation, and clonogenicity under normoxia and hypoxia. AR activity was measured using an androgen response element driven reporter assay and qPCR to measure expression of AR target genes. Xenograft-bearing mice were treated with SB203580 to measure tumor growth and serum prostate specific antigen (PSA). Results: Our results indicate that p38 MAPK and Hsp27 are activated under normoxia and hypoxia in response to androgens in CRPC cells. p38 MAPK inhibition diminished Hsp27 activation and the hypoxia-mediated increase in AR activity. Additionally, inhibition of p38 MAPK activity decreased proliferation and survival of CRPC cells in vitro and prolonged the survival of tumor-bearing mice. Conclusions: These results suggest that p38 MAPK inhibition may represent a therapeutic strategy to disrupt AR signaling in the heterogeneous CRPC tumor microenvironment.

## 1. Introduction

Prostate cancer is the second most frequently diagnosed cancer in men worldwide [1]. Androgens drive the growth of prostate cancer through androgen receptor (AR) signaling. As such, androgen deprivation therapy (ADT) blocks the activation of AR and is used for the treatment of prostate cancer. However, 10–20% of all patients treated with medical or surgical castration become resistant to androgen withdrawal, or develop “castration-resistant” prostate cancer (CRPC), within 5 years of follow-up [2]. Patients with CRPC are burdened with poor prognosis and decreased quality of life [3]. Aberrant androgen receptor signaling remains the major driver of castration-resistant prostate cancer (CRPC), rendering it a potential therapeutic target.

Low oxygenation (hypoxia) is a common feature of prostate cancer tumors that is associated with poor prognosis [4,5]. Hypoxia confers resistance to chemo-and radiation- therapy and selects for aggressive cancer cells that are able to adapt and survive in the nutrient and oxygen deficient microenvironment [6,7]. As such, it is important for novel therapeutics to be effective against both normoxic and hypoxic tumor cells. Hypoxia has been reported to induce AR signaling in hormone-sensitive LNCaP prostate cancer cells [8,9,10], but it remains unknown whether CRPC cells also utilize this signaling pathway and whether it confers a survival advantage in hypoxia.

A p38 mitogen-activated protein kinase (MAPK) and Heat shock protein 27 (Hsp27) driven signaling axis has emerged as an important regulator of AR activity in hormone-sensitive prostate cancer. p38 MAPK phosphorylates a wide range of proteins involved in the regulation of chromatin remodeling, gene expression, differentiation, survival, proliferation, apoptosis, and cell motility in response to cellular stress and cytokines [11,12]. The binding of androgen to AR triggers p38 MAPK-dependent phosphorylation of Hsp27, allowing Hsp27 to chaperone AR to the nucleus for transactivation of its target genes [13]. In the absence of androgens, alternative activation of AR by interleukin 6 also depends on p38 MAPK activity [14]. In line with this, p38 MAPK signaling was shown to be important for proliferation and survival of hormone-sensitive LNCaP cells [15,16,17]. Furthermore, neuroendocrine prostate cancer cells mediate docetaxel-resistance by secreting parathyroid hormone-related protein which increases AR activity in neighboring cells in a p38/Hsp27 dependent manner [18]. Lastly, p38 MAPK phosphorylation levels, which reflect its active state, correlate with prostate tissue progression from healthy tissue to neoplasia [19]. Current literature supports that p38 MAPK is activated in response to hypoxia in mouse embryo fibroblasts and human renal cells, ovarian cancer cells and hormone sensitive LNCaP prostate cancer cells [15,20,21,22]. In LNCaP cells, p38 MAPK/Hsp27 signaling has been proposed to be important for driving increased AR activity under hypoxia [15].

Given the urgent need for novel targeted agents in the treatment of CRPC and the emerging role of the p38 MAPK/Hsp27 signaling pathway in hormone-sensitive LNCaP cells, we sought to determine if CRPC cells are dependent on the p38/Hsp27 pathway for AR signaling. Since tumor hypoxia limits the efficacy of many cancer treatments, we also sought how oxygen availability affects this dependency. Here we show that p38 MAPK inhibition decreases cell proliferation, survival, and AR signaling in CRPC cells under normoxia and hypoxia and improves overall survival of mice bearing CRPC tumors.

## 2. Results

### 2.1. p38 MAPK Inhibition Decreases Cell Proliferation in Prostate Cancer Cells Expressing AR

**Figure 1:**
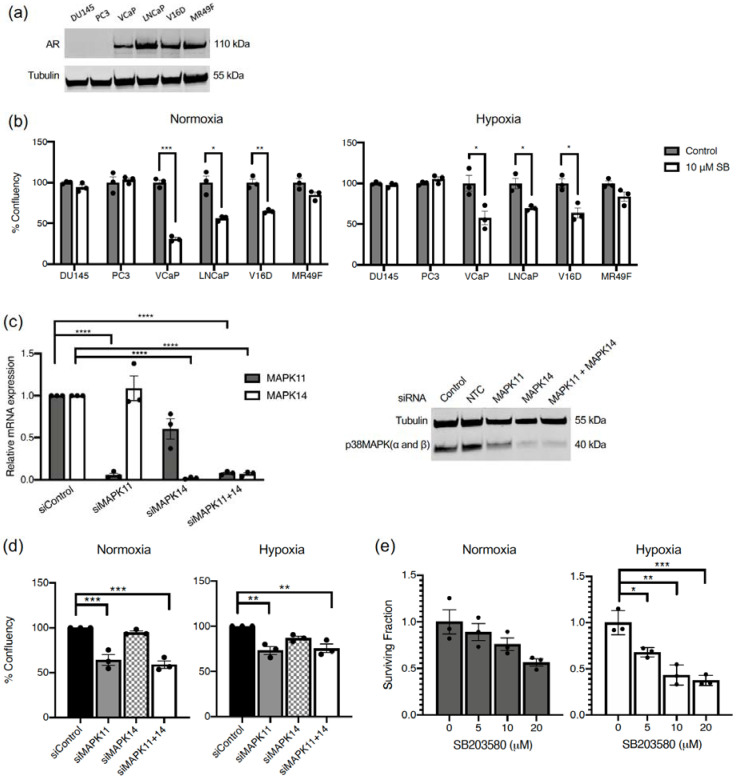
p38 MAPK inhibition decreases cell proliferation and survival in prostate cancer cells. (**a**) Western blots from DU145, PC3, VCaP, LNCaP, V16D and MR49F cells showing AR expression. (**b**) Indicated cell lines were treated with 10 µM SB203580 (SB) under normoxia (21% O_2_) (left) or hypoxia (0.2% O_2_) (right) for 150 h. Confluency was measured with the IncuCyte Live Cell Imaging system after confirming proportionality to cell numbers. Each data point represents an independent experiment, bars represent the mean value ± S.E.M. (**c**) V16D cells were transfected with siRNA targeting MAPK11, MAPK14, or non-targeting negative control (NTC). Expression of target genes was assessed by qPCR (left) or western blotting (right). (**d**) Cells as in (c) were placed in normoxia (21% O_2_) or hypoxia (0.2% O_2_) for 72 h and confluency was measured with the IncuCyte Live Cell Imaging system. (**e**) V16D cells were treated with various doses of SB203580 (5, 10, 20 µM) for 72 h under normoxia (21% O_2_) or hypoxia (0.2% O_2_). Single cells were seeded for clonogenic survival in triplicate and surviving fraction calculated from colony formation 14 days later. Data points represent independent experiments and bars represent the mean value ± S.E.M. (*: *p* ≤ 0.05; **: *p* ≤ 0.01; ***: *p* ≤ 0.001).

The role of p38 MAPK-mediated Hsp27 activation for AR nuclear translocation has been previously reported in hormone-sensitive LNCaP cells [13]. Therefore, we proposed that p38 MAPK inhibition would decrease cell proliferation in prostate cancer cells that are dependent on AR signaling for survival. To investigate this, a panel of prostate cancer cells were treated with the p38 MAPK inhibitor, SB203580 (SB), under normoxia and hypoxia. The panel contained AR-null cells (DU145, PC3) and AR-positive cells (VCaP, LNCaP, V16D, MR49F) (Figure 1a). The p38 MAPK inhibitor decreased cell proliferation under both normoxia and hypoxia in AR positive prostate cancer cells (VCaP, LNCaP, V16D), but not in AR-null prostate cancer cells (DU145, PC3) (Figure 1b). There was also a trend towards decreased in cell proliferation in the presence of SB203580 in the enzalutamide resistant MR49F cells, but this was not statistically significant. This indicates that p38 MAPK inhibition decreases cell proliferation in prostate cancer cells expressing AR.

To investigate whether SB203580 inhibition is on-target and which p38 isoform might be more important for the effect, *MAPK11* (p38β MAPK) and *MAPK14* (p38α MAPK) were transiently depleted with small interfering RNA (siRNA) in V16D cells. We focused on V16D since it represents CRPC and was sensitive to p38 MAPK targeting. Both siRNAs were highly efficient in depleting their target gene, while *MAPK14* siRNA also had a small effect on *MAPK11* expression on mRNA (left) and protein (right) level (Figure 1c). Nevertheless, siRNA-mediated knockdown with *MAPK11* alone phenocopied the results obtained with SB203580 by significantly decreasing cell proliferation under normoxia and hypoxia (Figure 1d). siRNA-mediated knockdown of *MAPK14* alone resulted in a smaller and non-statistically significant decrease in cell proliferation under normoxia or hypoxia, while combined siRNA knockdown of *MAPK11* and *MAPK14* gave similar results as *MAPK11* knockdown alone (Figure 1d). As such, *MAPK11* appears to drive the growth inhibitory phenotype of dual *MAPK11* and *MAPK14* inhibition and be important for cell proliferation.

To assess whether acute inhibition of proliferation translated into a decrease in long term cell survival, we treated cells with SB203580 under normoxia and hypoxia for 3 days and then plated the cells for colony formation in the absence of drug. SB203580 reduced survival in a dose dependent manner under hypoxia (Figure 1e). A similar trend, but less strong and not statistically significant (*p* = 0.06 at 20 µM) was observed in normoxia, possibly because the drug exposure was shorter than in the proliferation assay (Figure 1b). This result demonstrates that hypoxia offers no protection against the toxic effects of p38 inhibition, which is of importance for efficacy in the tumor microenvironment.

### 2.2. p38 MAPK Inhibition Decreases Hsp27 Phosphorylation, AR Activity and Expression of AR Target Genes under Normoxia and Hypoxia

**Figure 2:**
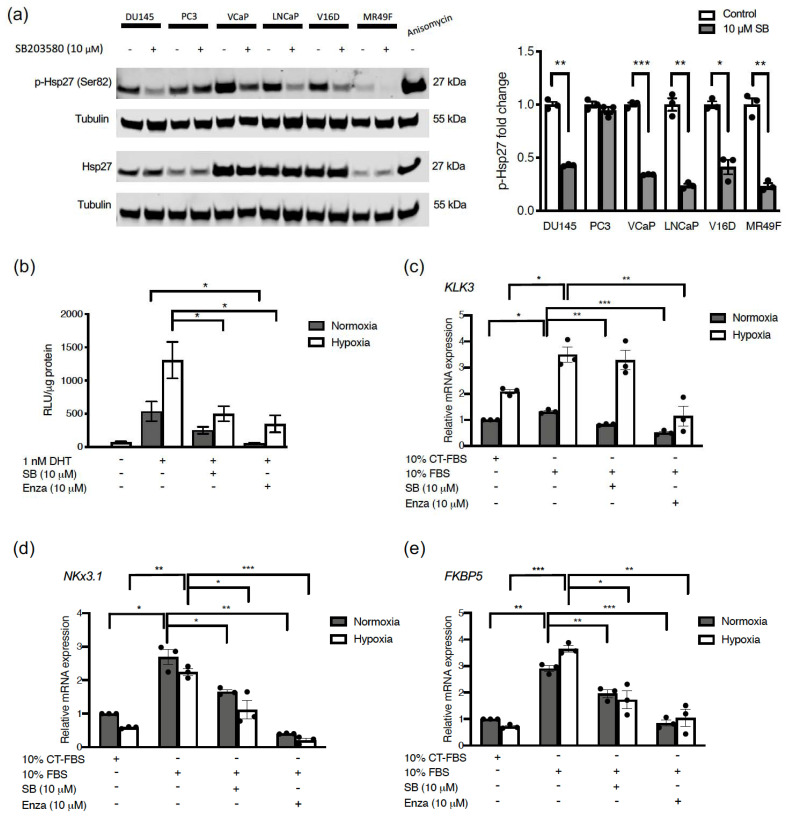
p38 MAPK inhibition decreases Hsp27 phosphorylation, AR activity and expression of AR target genes under normoxia and hypoxia. (**a**) Prostate cancer cell lines (DU145, PC3, VCaP, LNCaP, V16D, MR49F) were treated with 10 µM SB203580 for one hour and subjected to western blotting (left). V16D cells were treated with 25 µM of anisomycin for one hour as a positive control for p38 MAPK activation. Densitometry analysis was performed to calculate fold change of p-Hsp27 (Ser82) relative to Hsp27 for each cell line. (**b**) V16D cells were transfected with a plasmid encoding luciferase driven by the AR promoter. 24 h later, cells were treated with 1 nM DHT, 10 µM SB203580, or 10 µM enzalutamide (Enza) in 10% charcoal treated (CT)-FBS media (androgen depleted) and incubated under normoxia (21% O_2_) or hypoxia (0.2% O_2_) for 48 h. Relative light units (RLU) from luciferase was measured after addition of luciferin. (**c–e**) V16D cells were treated with 10 µM SB203580, or 10 µM enzalutamide for 48 h and relative mRNA expression of *KLK3* (**c**), *NKx3.1* (**d**), and *FKBP5* (**e**) was measured by RT-qPCR normalized to the average of *HPRT1* and *GUSB* gene expression. Fold change was calculated relative to the 10% CT-FBS negative normoxia control. Data points represent an independent experiment and bars represent the mean value ± S.E.M. (*: *p* ≤ 0.05; **: *p* ≤ 0.01; ***: *p* ≤ 0.001).

We sought to determine if Hsp27 phosphorylation is governed by p38 MAPK activity in the panel of cell lines. Indeed, SB203580 substantially reduced Hsp27 phosphorylation in all cell lines except PC3 (Figure 2a). Increasing the SB203580 dose made no difference to the levels of basal Hsp27 phosphorylation in PC3 cells (Figure S1). However, SB203580 treatment prevented Hsp27 phosphorylation induced by the p38 activating drug anisomycin (Figure S1). These results suggest that other kinases govern the basal phosphorylation status of Hsp27 in PC3 cells, but induction of p38 activity by stress can still be blocked by SB203580. In most cells however, it appears that basal levels of Hsp27 phosphorylation are driven by p38 activity (Figure 2a).

Since p38 MAPK mediated Hsp27 activity has previously been shown to be involved in AR translocation in castration sensitive LNCaP cells under normal oxygen conditions [13], we next aimed to determine if p38 MAPK also regulates AR signaling in CRPC cells and under hypoxic conditions. To this end, we transfected V16D cells with a luciferase reporter construct which is driven by the *KLK3* (encoding Prostate Specific Antigen (PSA)) partial promoter known to be responsive to AR binding. We performed experiments in the presence of castrate levels of dihydrotestosterone (DHT) and used enzalutamide as a positive control for AR signaling inhibition. Notably, AR activity was detected under hypoxic conditions even in the absence of DHT and remained higher than in normoxia in all treatment groups (Figure 2b). As would be expected from these CRPC cells, the addition of low levels of DHT substantially stimulated AR activity (Figure 2b). SB203580 treatment could reduce AR activity in hypoxia, similar to effects of enzalutamide. To determine if p38 MAPK inhibition also prevented endogenous AR activity, we measured expression of AR target genes *KLK3*, *NKx3.1* and *FKBP5* by qPCR. We compared gene expression in normoxia and hypoxia in the presence and absence of SB203580 in regular media (10% FBS). We included a reference condition of cells with charcoal-stripped (CT) FBS which provided minimal AR stimulation. mRNA expression of the endogenous *KLK3* gene was consistently higher in hypoxia than normoxia (Figure 2c), but SB203580 was largely ineffective at inhibiting its expression. Presumably, this reflects that AR-independent mechanisms can drive *KLK3* expression in these CRPC cells. In contrast to *KLK3*, SB203580 efficiently reduced expression of other AR-responsive genes such as *NKx3.1* (Figure 2d) and *FKBP5* (Figure 2e) in both normoxia and hypoxia. Taken together, these results indicate that p38 MAPK inhibition prevents AR activity under normoxia and hypoxia.

### 2.3. Hypoxia and AR Signaling Activate Hsp27

**Figure 3:**
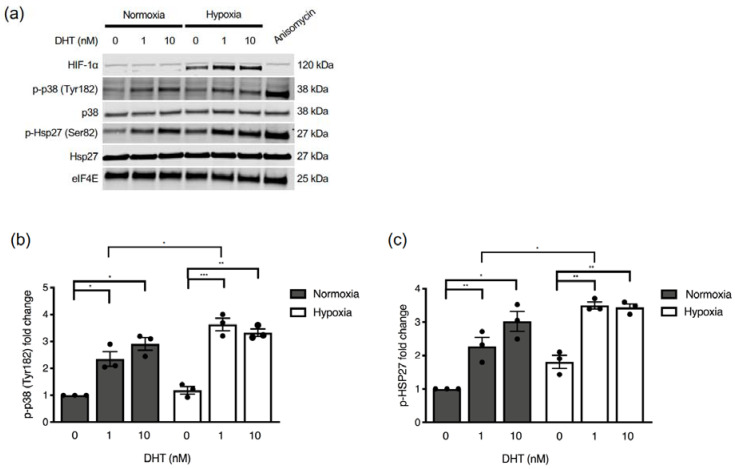
Androgen and hypoxia activate p38 MAPK and Hsp27. (**a**) V16D cells were exposed to 0 nM, 1 nM, or 10 nM DHT in charcoal-stripped media for 6 h in normoxia (21% O_2_) or hypoxia (0.2% O_2_). 25 µM of anisomycin for one hour was used as a positive control for p38 MAPK activation. Total cell lysates were subjected to western blotting. Densitometry analysis of protein bands was performed to calculate fold change of p-p38 (Tyr182) (**b**) and p-Hsp27 (Ser82) (**c**) relative to normoxia control with 0 nM DHT and normalized to eIF4E. Data points represent independent experiments and bars represent the mean value ± S.E.M. (*: *p* ≤ 0.05; **: *p* ≤ 0.01; ***: *p* ≤ 0.001).

We wondered if increased AR activity in CRPC cells in hypoxia (Figure 2b,c) could be due to hypoxia activating p38 MAPK and/or Hsp27. To investigate this, we assessed p38 MAPK activation as reflected by its Tyr182 phosphorylation, as well as Hsp27 phosphorylation, in normoxia and hypoxia in the presence of castrate levels of DHT. Data show that low DHT exposure (1nM) results in p38 MAPK activation, which is slightly higher in hypoxia than in normoxia (Figure 3a,b). This was completely mirrored in Hsp27 phosphorylation. However, hypoxia also stimulated Hsp27 phosphorylation in the complete absence of DHT and without accompanying p38 activation (Figure 3a,c, *p* = 0.054). Taken together, these results show that p38 MAPK is activated by low levels of androgen in CRPC cells, that this activation is exacerbated under hypoxic conditions, and an additional androgen- and p38 MAPK-independent mechanism for Hsp27 activation by hypoxia alone exists.

### 2.4. p38 MAPK Inhibition Prolongs Survival in Mice Bearing CRPC Xenografts

**Figure 4:**
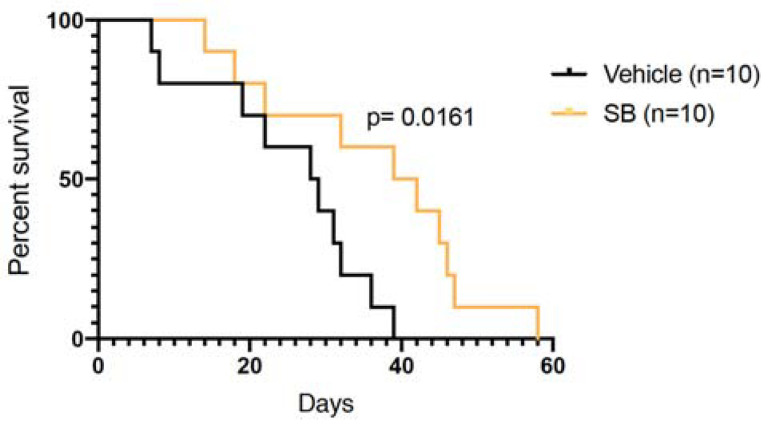
p38 MAPK inhibition prolongs survival of mice bearing CRPC xenografts. Subcutaneous V16D xenografts were established and treatment with vehicle or SB203580 (10 mg/kg) started when tumors reached 200 mm^3^, in a 5 days on and 2 days off schedule until endpoint. Kaplan-Meier survival curves are shown for the time to reach tumor volume endpoint (1000 mm^3^) from treatment start. Tumor growth curves from individual mice are shown in Figure S2a.

Since p38 MAPK inhibition by SB203580 could block Hsp27 phosphorylation, reduce expression of AR-driven gene expression and inhibit cell proliferation and survival in normoxia and hypoxia, we sought to determine whether SB203580 could also inhibit tumor growth in V16D xenografts. To this end, we established subcutaneous xenografts in immune compromised male mice, and treated them with SB203580 once tumors reached 200 mm^3^. SB203580 was well-tolerated and did not lead to any apparent clinical side-effects. Some mice reached endpoint early in the study, precluding comparisons of average tumor volumes in the two treatment groups (Figure S2a). However, SB203580 treatment significantly prolonged survival of tumor bearing mice (Figure 4). During treatment, we also collected blood for serum PSA measurements as a reflection of AR activity and tumor burden. Although PSA levels were highly heterogeneous, we observed that over the last week of life, only 1 out of 8 mice in the SB203580 treated group had a >1.5-fold increase in PSA levels, compared to 5 out of 8 in the control group (Figure S2b) (*p* = 0.07). Taken together, our results demonstrate that SB203580 can slow growth of CRPC xenografts.

## 3. Discussion

Androgen deprivation therapy (ADT) is a fundamental pillar of prostate cancer treatment, but resistance develops in ~20% of patients. In spite of such castration resistance, cells often still depend on AR signaling, which can be stimulated through other mechanisms and be hyper-sensitive to low androgen levels [23]. Tumor hypoxia is associated with progression to CRPC, AR signaling, and treatment resistance [5,10,24]. As such, targeting the AR signaling cascades that are utilized under normoxia and hypoxia is a potential new therapeutic strategy in CRPC.

Here, we report for the first time that CRPC cells utilize the p38 MAPK/Hsp27 pathway for AR signaling under normoxia and hypoxia. Notably, we find that in CRPC cells, very low (castrate) levels of androgen are sufficient to stimulate p38 MAPK activity. As such, this pathway which is operative in hormone-sensitive LNCap cells [13] has acquired hyper-sensitivity to androgens to maintain activity during the development of castration-resistance and remains a potential therapeutic target in this context. We also report here that p38 activity is further exacerbated by hypoxia in CRPC cells, and drives Hsp27 activation, AR signaling, cell proliferation, cell survival and tumor growth (see graphical abstract). However, hypoxia appears to additionally stimulate Hsp27 activation independent of androgens and p38 MAPK and drives expression of some canonical AR-responsive genes in the presence of p38 MAPK inhibition. As such, hypoxia stimulates Hsp27/AR signaling both in an androgen/p38-dependent and -independent manner (see graphical abstract) in CRPC.

In spite of this, we found p38 MAPK inhibition to be equally effective at reducing cell proliferation and survival under normoxic and hypoxic conditions. This suggests that although p38 MAPK-independent effects of hypoxia can be observed on a biochemical level and may contribute to other phenotypes than those measured here, it is the p38 MAPK-dependent effects that are most dominant for growth and survival. As such, we conclude that inhibition of the p38 MAPK/Hsp27/AR signaling axis may represent a novel strategy for targeting CRPC. Our data suggest that only patients carrying AR positive CRPC tumors would be expected to respond to p38 MAPK inhibition. In line with the model (see graphical abstract), AR negative cell lines PC3 and DU145 did not respond to p38 inhibition (Figure 1b). We also note that overall Hsp27 expression was low in AR null cell lines DU145 and PC3, which might suggest that cells not dependent on AR signaling also have less need for Hsp27.

Much is still to learn about the mechanism governing anti-cancer effects of p38 MAPK inhibition in prostate cancer. The role of various p38 MAPK isoforms remain elusive, as one other study reported AR-inhibitory effects of p38α [25]. In our cells, data suggested that p38α is expressed but inconsequential for cell growth, while p38β appears to drive proliferation. Furthermore, the direct substrate for p38 MAPK that is mediating the effect on Hsp27 is not known, although a candidate is PRAK (MAPKAPK5) since it is both a kinase for Hsp27 and a known substrate of p38 MAPK [26,27,28]. A more complete understanding of the way that AR and hypoxia in concert govern gene expression would also be beneficial. Interestingly, a previous study of hormone-sensitive LNCaP cells suggested that p38 MAPK inhibition could decrease binding of the hypoxia-induced transcription factor HIF-1 to its promoter and thereby mitigate HIF-induced gene expression [15]. The same study indicated that p38 MAPK inhibition could prevent hypoxia-induced *stimulation* of proliferation, survival and invasion. In contrast, hypoxia inhibited proliferation of both hormone sensitive LNCaP cells and the castration resistant V16D cells in our study, while p38 MAPK inhibition further reduced proliferation in all conditions. The possible cooperation between the hypoxia-induced transcription factor HIF-1 and AR is complex and most certainly context-dependent [15,29,30,31].

A limitation of this study is the lack of biomarker assessment of response to SB203580 upstream of PSA in vivo. Both tumor growth and PSA data suggest inter-tumor heterogeneity in SB203580 response, and it is unclear whether this heterogeneity relates to drug delivery due to differences in e.g., vascularization, or other factors. Indeed, spontaneous tumor hemorrhage was intermittently observed in this study, suggesting the presence of immature and leaky tumor vasculature. Furthermore, all molecularly targeted agents may have pleiotropic effects that can affect response, and the contribution of such factors are difficult to rule out. Future studies should aim to monitor tumor drug concentrations as well as response biomarkers such as p-Hsp27 in addition to PSA. It would also be interesting to assess the effect of SB203580 on tumors knockout for various p38 isoforms to fully delineate potential on- and off-target drug effects in vivo. Nevertheless, data presented here represent proof-of-principle on which we can continue to build for potential clinical translation.

Also, it would be interesting to explore the effect of other, clinically available, p38 inhibitors. In a randomized phase 1b/2 trial in recurrent ovarian cancer, patients that received the p38 MAPK inhibitor ralimetinib in addition to gemcitabine and carboplatin, had a modest improvement in progression free survival [32]. Ralimetinib is also being explored in other disease sites with other combination therapies [33,34]. Given the specific potential for p38 MAPK inhibition to affect androgen signaling shown here and elsewhere, there may be an opportunity to explore this drug for the treatment of prostate cancer. Since effective first-line treatments exist for many prostate cancer risk groups upon presentation, the key may be to identify patients of high risk for disease progression and/or patients that have progressed to CRPC.

Furthermore, since the efficacy of both radiotherapy and chemotherapy is hampered by hypoxia, p38 MAPK inhibition could represent an alternative or complementary strategy for patients carrying hypoxic tumors. Whether this would be more attractive than current second line anti-androgen treatments remains to be seen, and would probably depend on toxicity profiles and co-morbidities. On that note, p38 is well-known to regulate inflammation, and a few studies have suggested that p38 inhibition might mitigate radiation-induced gene expression associated with promotion of inflammation [35,36,37]. It would be interesting to assess whether p38 inhibition thereby could offer radioprotection of normal tissues and further widen the therapeutic window in the context of radiotherapy.

In conclusion, we show here for the first time that p38 MAPK and Hsp27 are activated by castrate levels of androgens in CRPC cells under normoxia, and even more so in hypoxia. However, hypoxia also drives additional Hsp27 activation and AR-linked gene expression independent of p38 MAPK. Nevertheless, p38 MAPK inhibition represents a strategy to inhibit the proliferation and survival of AR-dependent prostate cancer cells in vitro and in vivo, including those representing CRPC.

## 4. Materials and Methods

### 4.1. Cell Culture

DU145, LNCaP, MR49F, and V16D cells were cultured in RPMI-1640 medium. MR49F and V16D cells were kindly provided by Dr. Amina Zoubeidi (Vancouver Prostate Centre, University of British Columbia, BC, Canada) [38]. VCaP and PC3 cells were cultured in DMEM and MEM medium, respectively. All cells were passaged in media containing 10% FBS (Gibco^TM^, Thermo Fisher Scientific, Waltham, MA, USA). Additionally, V16D and MR49F cells are supplemented with 1 mM sodium pyruvate. MR49F cells were maintained in media with 10 μM enzalutamide.

### 4.2. Chemicals

SB203580 (4-(4-(4-fluorophenyl)-2-(4-(methylsulfinyl)phenyl)-1H-imidazol-5-yl)pyridine) (SelleckChem, Houston, TX, USA), anisomycin (Sigma Aldrich, St. Louis, MO, USA) and 5α- dihydrotestosterone (Sigma Aldrich) were dissolved in DMSO, DMSO and methanol respectively.

### 4.3. Cell proliferation Assay

IncuCyte^®^ ZOOM Live-Cell Imaging system (Essen Bioscience, Ann Arbor, MI, USA) was used for monitoring cell proliferation. Cells were seeded at 1000 to 5000 cells/well in 96-well plates. 24 h after seeding, cells were treated with 0–10 μM of SB203580, 10 μM enzalutamide, or DMSO control and incubated in normoxia (21% O_2_) or hypoxia (0.2% O_2_) in a H35 HypOxystation (Don Whitley Scientific, Frederick, MD, USA). The plate was scanned, and phase-contrast images used for calculation of confluency. All cell lines grew as monolayers with confluence being proportional to cell number.

### 4.4. Western Blotting

Total protein was extracted using RIPA lysis buffer (Tris-HCl: 50 mM, pH 7.4; NP-40: 1%; Na-deoxycholate: 0.25%; NaCl: 150 mM; EDTA: 1 mM), supplemented with a 1 × protease and phosphatase inhibitor (Thermo Fisher Scientific). Protein concentrations were quantified by Bicinchoninic Acid (BCA) Protein Assay kit (Thermo Fisher Scientific) and the FLUOstar Omega Microplate (BMG LABTECH, Ortenberg, Germany) reader by measuring absorbance at 560 nm. Lysates were boiled with Bolt^TM^ lithium dodecyl sulphate (LDS) Sample Buffer (Thermo Fisher Scientific) and 20–30 μg of protein lysate underwent electrophoresis on Bolt^TM^ 4–12% Bis-Tris Plus Gels. Proteins were transferred to a polyvinylidene difluoride membrane (Thermo Fisher Scientific) using the Mini Trans-Blot^®^ Electrophoretic Transfer Cell (Bio-Rad, Hercules, CA, USA). Membranes were blocked with Odyssey blocking buffer (LI-COR Biosciences, Lincoln, NE, USA) for 30 min at room temperature. The membrane was incubated with antibodies diluted in Odyssey blocking buffer (LI-COR Biosciences) overnight at 4 °C to detect the following proteins: p-p38 MAPK (Tyr182; E-1; 1:500; Santa Cruz Biotechnology, Dallas, TX, USA), p38 MAPK (1:1000), p-Hsp27 (Ser82; 1:1000), Hsp27 (G31; 1:1000), AR (D6F11; 1:1000), HIF-1ɑ (1:1000; BD Biosciences, San Jose, CA, USA), eIF4E (1:1000; BD Biosciences), and beta-tubulin (1:10,000; Abcam, Cambridge, UK). Antibodies were obtained from Cell Signaling Technology (Danvers, MA, USA) unless otherwise indicated. Blots were incubated with IRDye 680RD and/or IRDye 800CW secondary antibodies (1:10,000; LI-COR Biosciences) and imaged using LI-COR Odyssey imaging system (LI-COR Biosciences) for immunofluorescent western blot analysis. Protein bands were quantified using Image Studio (LI-COR Biosciences). Original blots can be found at Supplementary file 1.

### 4.5. AR Activity Luciferase Assay

The pENTR-GAL4RE-FL, pENTR-PSEBC-Gal4vp16, and pRL-null plasmids were kindly gifted by Dr. Frédéric Pouliot (Laval University, QC, Canada) [39]. V16D cells were transiently transfected with 100 ng pENTR-GAL4RE-FL plasmid, 100 ng pENTR-PSEBC-Gal4vp16 plasmid and 100 ng of Renilla luciferase expression vector pRL-null as a transfection control, using Lipofectamine 2000 transfection reagent according to the manufacturer’s instructions (Thermo Fisher Scientific). 24 h after transfection, the media was replaced, and cells were incubated in normoxia (21% O_2_) or hypoxia (0.2% O_2_) for 48 h. Cell lysates were prepared by direct lysis at room temperature using the Passive Lysis Buffer (Promega, Madison, WI, USA). Luciferase activity was measured using the Dual luciferase assay kit according to the manufacturer’s instructions (Promega) and read using FLUOstar Omega Microplate reader (BMG LABTECH).

### 4.6. RNA Extraction and Quantitative PCR

Total RNA was extracted using the RNeasy Mini Kit (Qiagen, Hilden, Germany). Isolated RNA was treated with DNase I (Invitrogen, Thermo Fisher Scientific) to eliminate DNA contamination. Extracted RNA was reverse transcribed using qScript cDNA SuperMix (Quantabio, Beverly, MA, USA) to produce cDNA according to manufacturer’s instructions. qPCR was performed using predesigned Taqman Gene Expression assays for the genes: *MAPK11, MAPK14, KLK3*, *NKx3.1*, *FKBP5*, *HPRT1*, and *GUSB* (Applied Biosystems, Foster City, CA, USA). *HPRT1* and *GUSB* were used as housekeeping controls. Quantitative PCR was performed using the Eppendorf Mastercycler ep Gradient S PCR System and Taqman Fast Advanced Master Mix (Applied Biosystems). Relative fold change was calculated using the 2^−ΔΔCt^ method.

### 4.7. Clonogenic Assay

Cells were treated with 0, 5, 10, 20 μM SB203580 and incubated under normoxia (21% O_2_) or hypoxia (0.2% O_2_) for 3 days. Cells were trypsinized, plated as single cells and incubated under normoxia in the absence of drug for 14 days to assess colony formation. Cells were fixed and stained using 0.2% (*w*/*v*) methylene blue in 80% ethanol. Colonies containing more than 50 cells were scored as a surviving colony. Plating efficiency (PE) was calculated for the untreated DMSO control as: PE = (average # of colonies formed/ # of cells seeded). Surviving fraction (SF) was calculated as: SF = (PE of treatment)/(PE of untreated).

### 4.8. siRNA Transfections

Cells were transfected in 6-well plates with 10 nM MAPK11 (s11155), 10 nM MAPK14 (s3585) siRNAs (Thermo Fisher Scientific), or 10 nM Stealth RNAi siRNA Negative Control (Invitrogen). All transfections were conducted using standard forward transfection protocols using Lipofectamine RNAiMAX Transfection Reagent (Invitrogen) according to manufacturer’s instructions.

### 4.9. Xenograft Experiments

All animal experiments were performed according to operating procedures approved by the Princess Margaret Cancer Centre Animal Care Committee, aligned with guidelines from the Canadian Council on Animal Care. Treatment groups of *n* = 10 were selected based on a requirement for 80% power to resolve a 35% difference in tumor volume with standard deviation of 33% and *p* = 0.05. Male NOD-*scid* IL2Rgamma^null^ mice were injected subcutaneously in the flank with 2 × 10^6^ V16D cells (1:1 suspension in Matrigel). The length and width of tumors were measured 2–3 times/week using calipers. Tumor volume was calculated by (length × width^2^)/2. Once tumors reached 200 mm^3^, mice were randomly assigned to (i) vehicle (5% DMSO, 30%, PEG 300, 5% Tween 80) or (ii) SB203580 (10 mg/kg in the same vehicle) (Supplementary Table S1). Mice were treated via intraperitoneal (i.p) injection 5 times per week (Mon–Fri) until they reached endpoint at tumor volume 1000 mm^3^. Whole blood (100 uL) was collected and was centrifuged at 2000× *g* for 10 min at 4 °C to collect approximately 50 μL of plasma. Serum PSA was measured using a quantitative human PSA enzyme-linked immunosorbent assay (ELISA) kit (Anogen, Mississauga, ON, Canada) as per the manufacturer’s protocol.

### 4.10. Statistical Analysis

Unless otherwise specified, statistical analysis was performed using the two-sided t-test with Welch’s correction and two-way analysis of variance (ANOVA) with Dunnett’s Multiple Comparison correction on GraphPad Prism v8. Statistical analysis was performed for the Kaplan-Meier survival curves using the Gehan-Breslow-Wilcoxon test. Error bars represent standard error of the mean (SEM).

## 5. Conclusions

In conclusion, we propose that p38 MAPK is an attractive therapeutic target for preventing transactivation of AR in CRPC cells under normoxia and hypoxia. Here, we demonstrate that inhibition of p38 MAPK decreases the growth and survival of prostate cancer cells that are dependent on AR signaling. This inhibition is effective in both normoxia and hypoxia, including in cells representative of CRPC. We demonstrate that the p38β MAPK isoform drives proliferation in CRPC cells. We also show that p38 MAPK activity promotes Hsp27 phosphorylation, which has been previously shown to chaperone AR to the nucleus. P38 MAPK activity, Hsp27 phosphorylation and AR activity is enhanced by low (castrate) levels of androgen in CRPC cells, and further exacerbated by hypoxia. As such, p38 MAPK inhibition significantly reduced expression of AR-driven genes under normoxia and hypoxia. SB203580 treatment significantly prolonged the survival of mice bearing CRPC tumors. This is the first study to investigate the p38 MAPK/Hsp27 signaling pathway in facilitating AR transactivation in CRPC cells under normoxia and hypoxia. Inhibiting p38 MAPK may be an attractive therapeutic target for the treatment of CRPC.

## Data Availability

Data is contained within this article and supplementary material.

## Supplementary Materials

The following are available online at https://www.mdpi.com/2072-6694/13/4/831/s1, Figure S1: PC3 cells were treated with SB203580 (10 μM or 50 μM), 25 μM anisomycin, or the combination for one hour and subjected to western blotting (left). Densitometry analysis of protein bands was performed on Image Studio to calculate fold change of p-Hsp27 (Ser82) relative to tubulin (right). Bars represent the mean of three independent experiments ± S.E.M (*: *p* ≤ 0.05), Figure S2: (a) Subcutaneous V16D xenografts were established and treatment with vehicle or SB203580 (10 mg/kg) started when tumors reached 200 mm^3^ (Day 0), in a 5 days on and 2 days off schedule until endpoint. Tumor growth curves from individual mice are shown normalized to their starting volume. (b) PSA was measured by ELISA in blood samples from tumor-bearing mice. Fold change in PSA for each mouse in their last week of life is plotted for each treatment group. A Mann-Whitney test was used to obtain the displayed *p*-value, Table S1: Tumor volume and endpoint characteristics for treatment groups. Supplementary file 1 for original images of western blots.
