# Supplementary material for: p38 MAPK Inhibition Mitigates Hypoxia-Induced AR Signaling in Castration-Resistant Prostate Cancer
Source: Cancers (Basel). 2021 Feb 17;13(4):831. doi: 10.3390/cancers13040831 (PMC7922949; doi:10.3390/cancers13040831)
Supplement: Supplementary file 1 [file cancers-13-00831-s001.zip › cancers-1098619-original-images.pdf]

Figure 1a

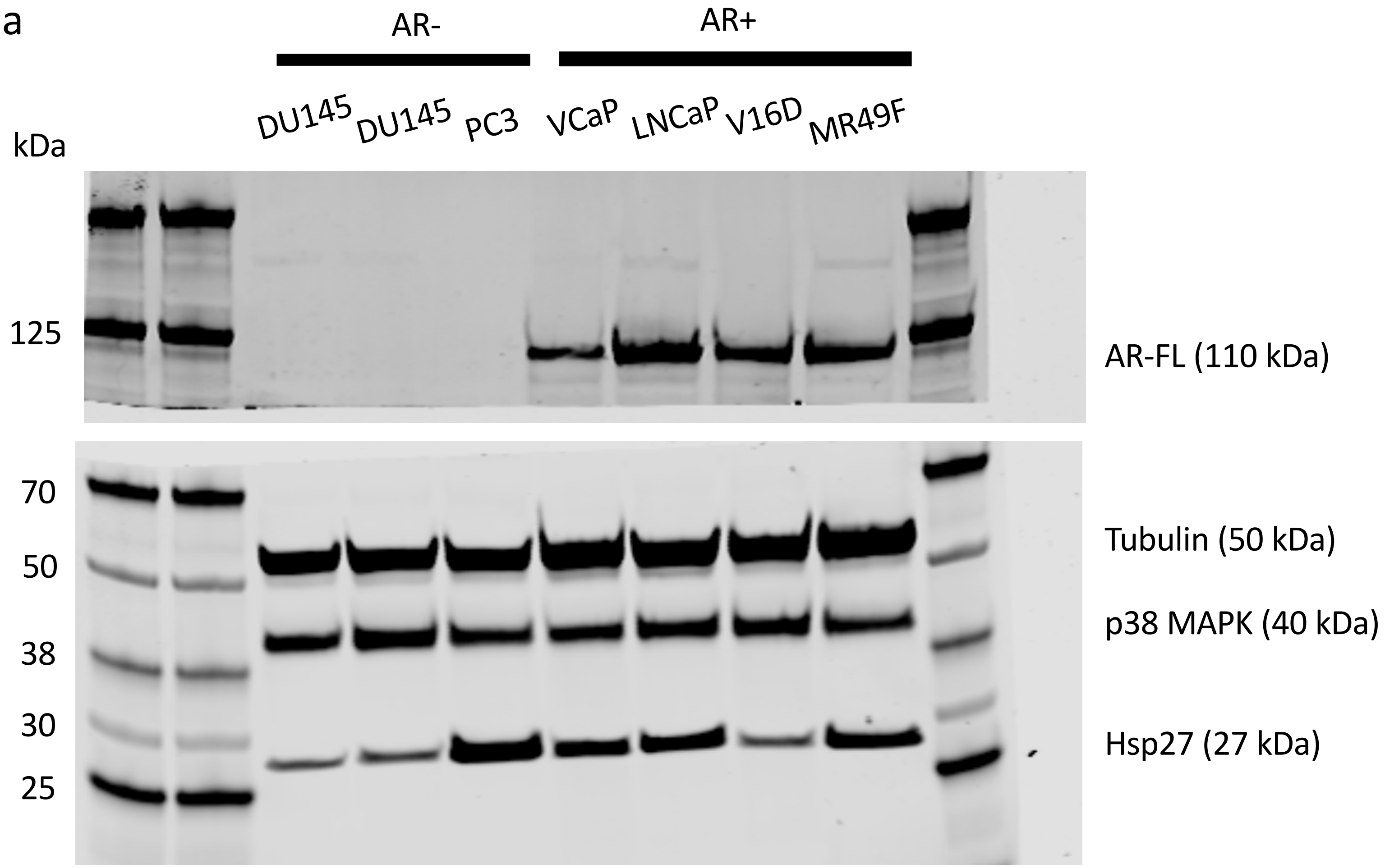

Cut blot from the same gel

Figure 1c

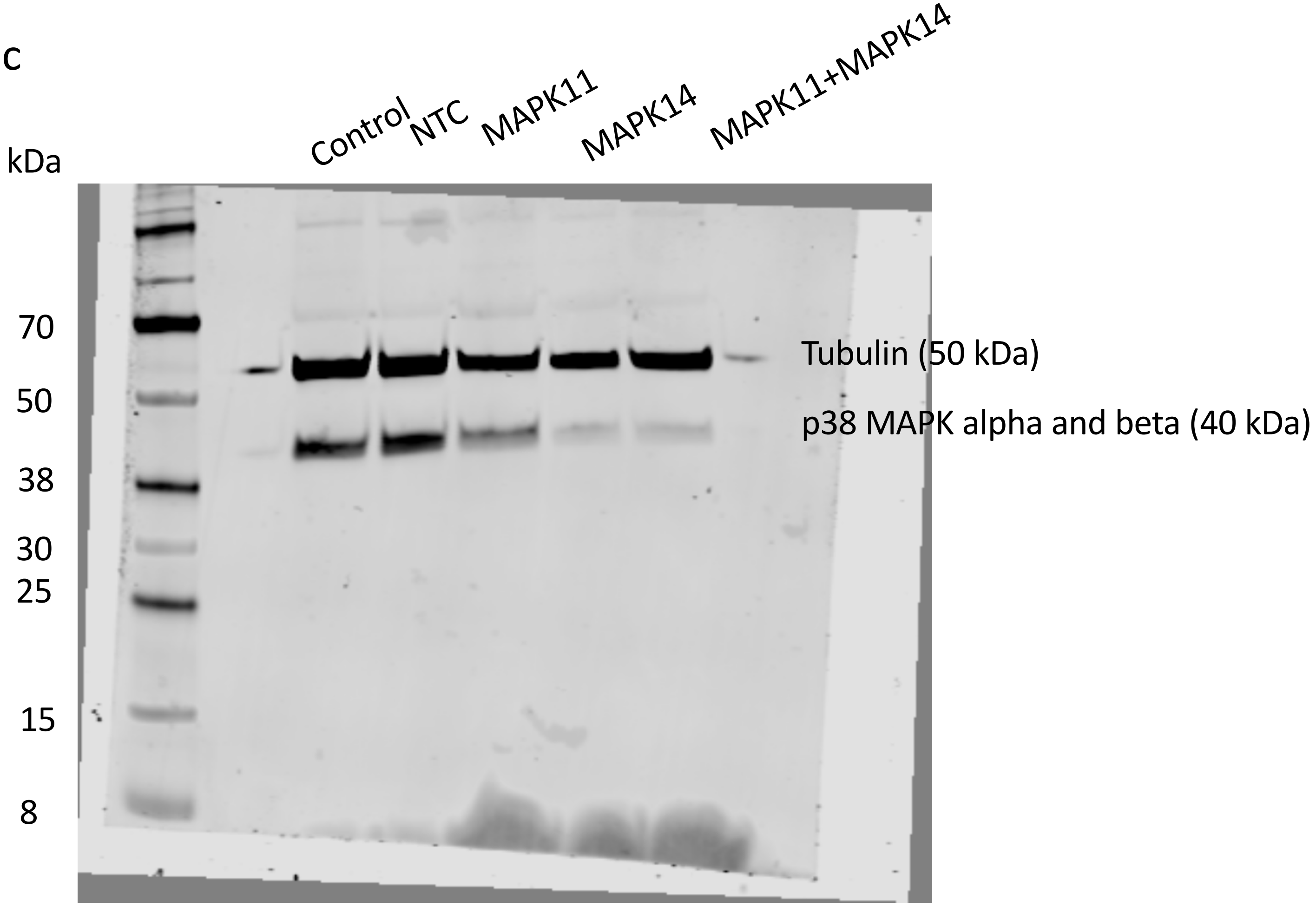

kDa
Control
NTC
MAPK11
MAPK14
MAPK11+MAPK14
70
50
38
30
25
15
8
Tubulin (50 kDa)
p38 MAPK alpha and beta (40 kDa)

Figure 2a

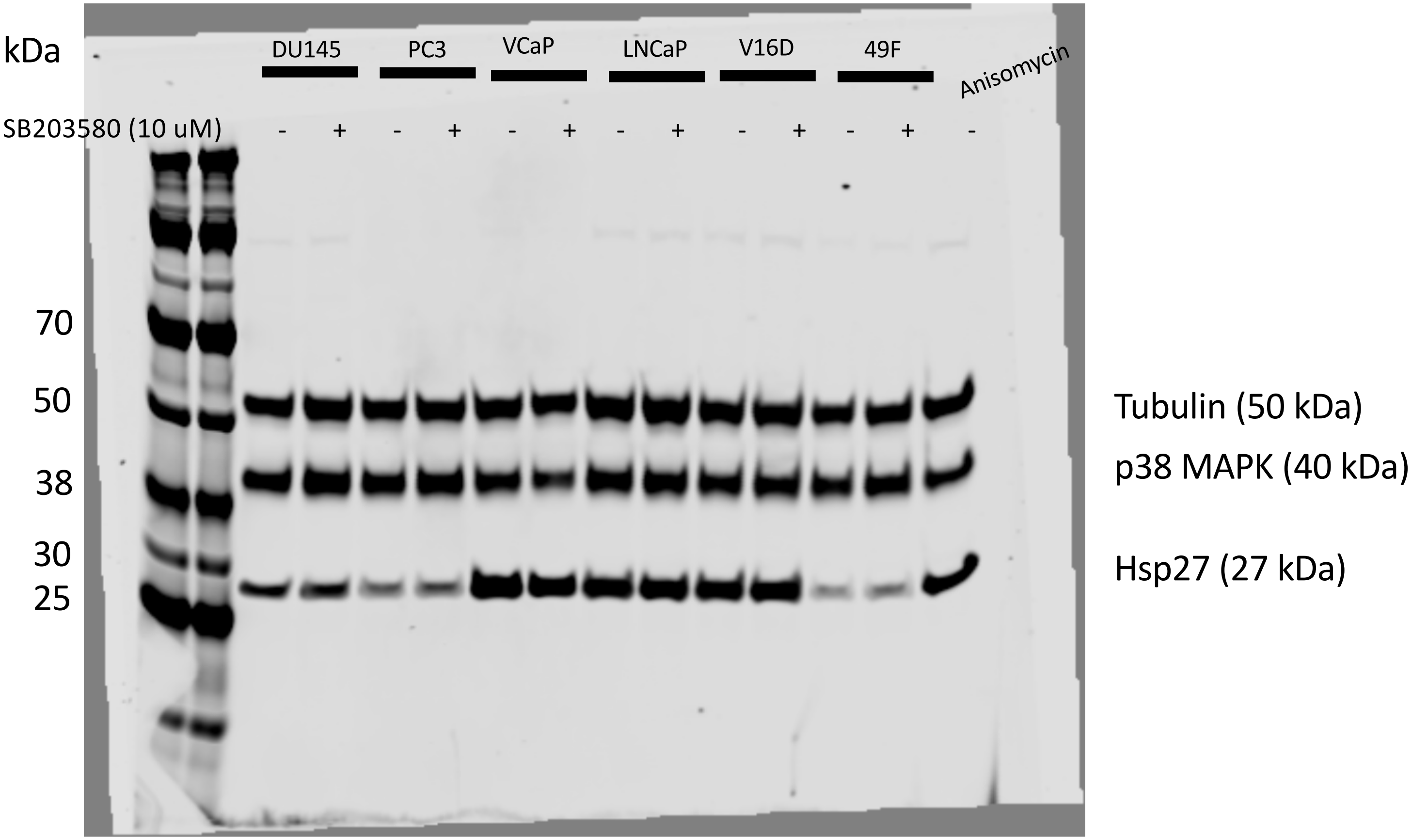

kDa
DU145
PC3
VCaP
LNCaP
V16D
49F
Anisomycin
SB203580 (10 uM)
- + - + - + - + - + - + -
70
50
38
30
25
Tubulin (50 kDa)
p38 MAPK (40 kDa)
Hsp27 (27 kDa)

Figure 2a (continued)

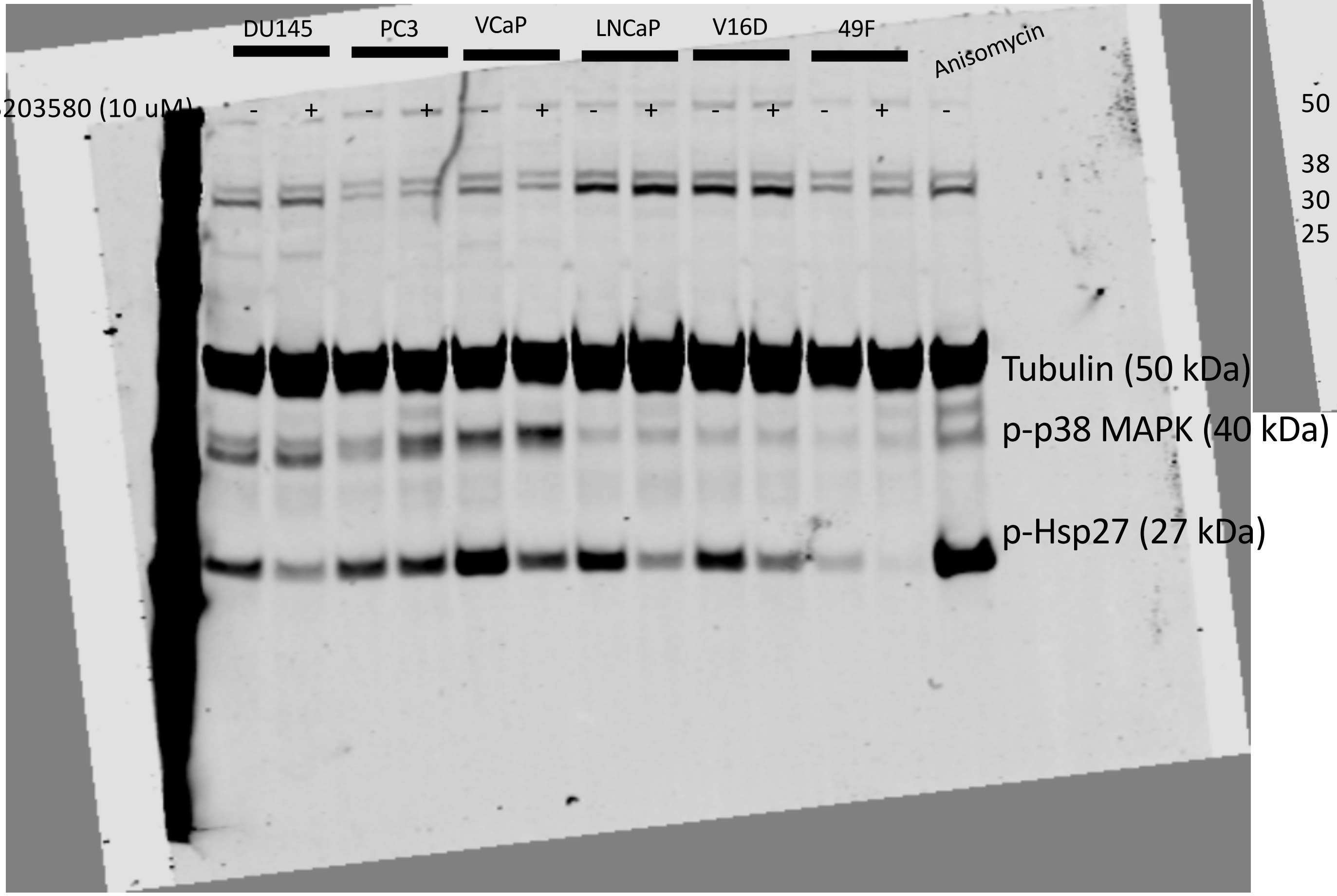

High exposure to visualize p-p38 and p-Hsp27

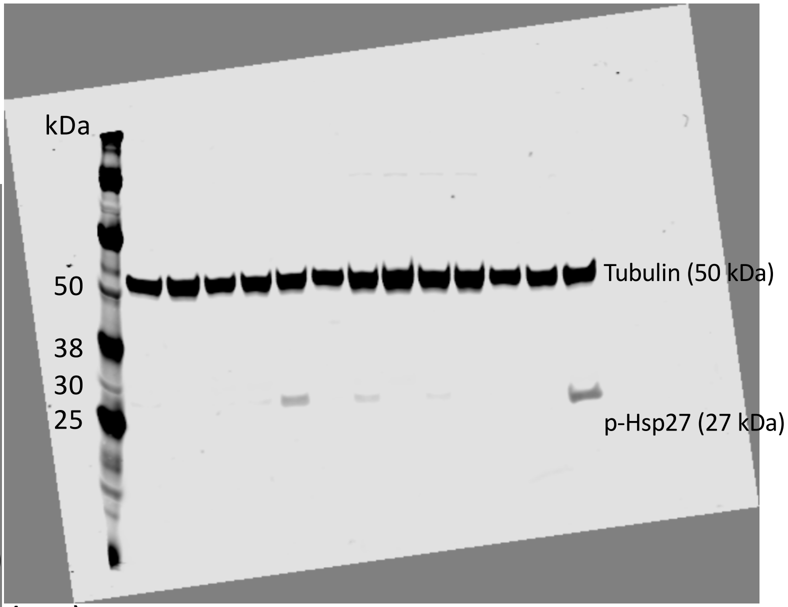

Same blot, lower exposure

Figure 3a

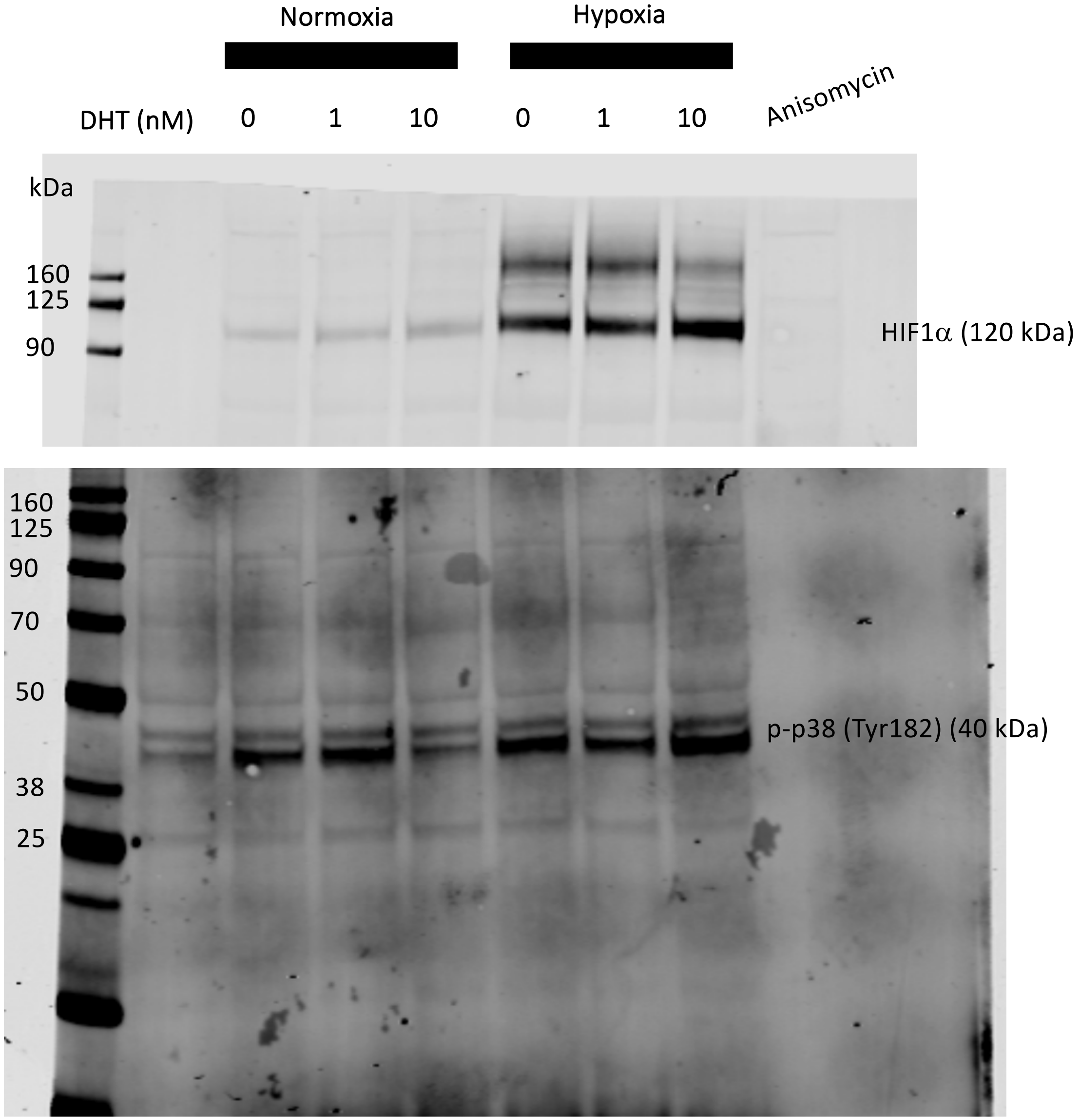
Normoxia
Hypoxia
DHT (nM)
0
1
10
0
1
10
Anisomycin
kDa
160
125
90
HIF1α (120 kDa)
160
125
90
70
50
38
25
p-p38 (Tyr182) (40 kDa)

Figure 3a

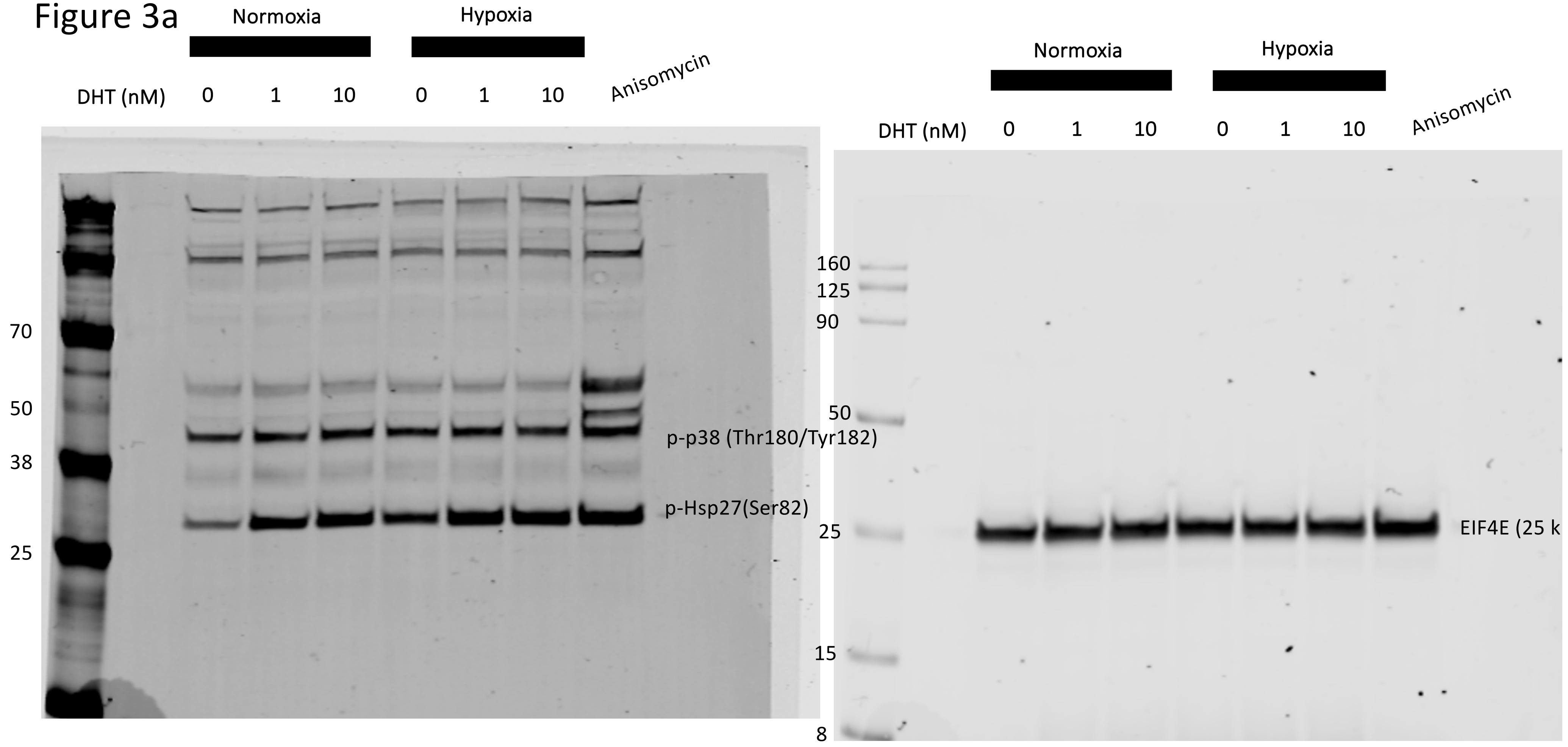
Normoxia
Hypoxia
DHT (nM)
0
1
10
0
1
10
Anisomycin
70
50
38
25
p-p38 (Thr180/Tyr182)
p-Hsp27(Ser82)
Normoxia
Hypoxia
DHT (nM)
0
1
10
0
1
10
Anisomycin
160
125
90
50
25
15
8
EIF4E (25 k
