# Supplementary material for: p38 MAPK Inhibition Mitigates Hypoxia-Induced AR Signaling in Castration-Resistant Prostate Cancer
Source: Cancers (Basel). 2021 Feb 17;13(4):831. doi: 10.3390/cancers13040831 (PMC7922949; doi:10.3390/cancers13040831)
Supplement: Supplementary file 1 [file cancers-13-00831-s001.zip › cancers-1098619-suppl.pdf]

Supplementary Figure 1

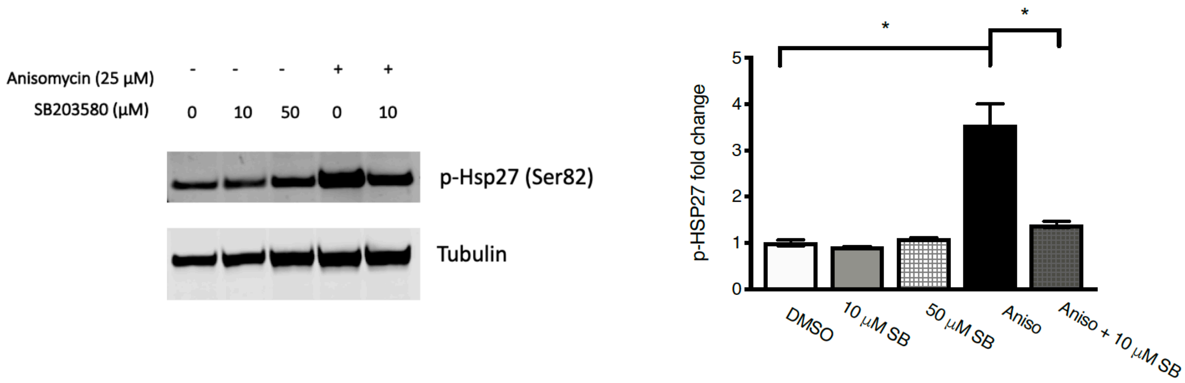

Supplementary Figure 1.
PC3 cells were treated with SB203580 (10 µM or 50 µM), 25 µM anisomycin, or the combination for one hour and subjected to western blotting (left). Densitometry analysis of protein bands was performed on Image Studio to calculate fold change of p-Hsp27 (Ser82) relative to tubulin (right). Bars represent the mean of three independent experiments ± S.E.M (*: $p \leq 0.05$).

Supplementary Figure 2

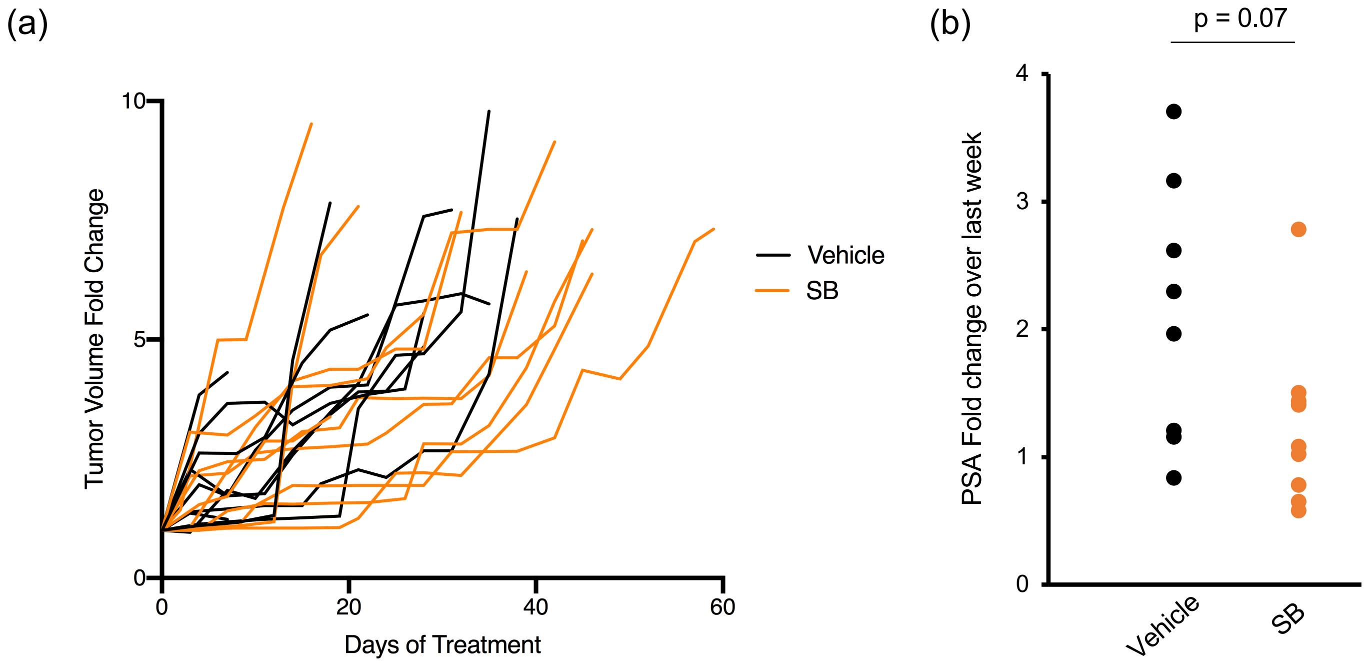

Supplementary Figure 2.
(**a**) Subcutaneous V16D xenografts were established and treatment with vehicle or SB203580 (10 mg/kg) started when tumors reached 200 mm^3 (Day 0), in a 5 days on and 2 days off schedule until endpoint. Tumor growth curves from individual mice are shown normalized to their starting volume. (**b**) PSA was measured by ELISA in blood samples from tumor-bearing mice. Fold change in PSA for each mouse in their last week of life is plotted for each treatment group. A Mann-Whitney test was used to obtain the displayed p-value.

Supplementary Table 1

	Vehicle (n=10)	SB203580 (n=10)
Average tumor volume at Day 0 [mm^3]	216.0	202.9
Standard Deviation of tumor volumes at Day 0 [mm^3]	39.3	26.6
Median tumor volume at Day 0 [mm^3]	200.6	191.3
Interquartile range of tumor volumes at Day 0 [mm^3]	38.7	43.0
Median number of days to endpoint	28.0	40.5
Interquartile range of days to endpoint	12.0	21.5

Supplementary Table 1.
Tumor volume and endpoint characteristics for treatment groups.
